# The significance of woody vegetation's nonproductive elements for the overwintering of key biocontrol agents in intensively used agricultural areas

**DOI:** 10.1002/ps.70188

**Published:** 2025-09-21

**Authors:** Jana Niedobová, Tomáš Kudláček

**Affiliations:** ^1^ Department of Forest Ecology, Faculty of Forestry and Wood Technology Mendel University in Brno Brno Czech Republic; ^2^ University of Greifswald Institute for Mathematics and Computer Science & Center for Functional Genomics of Microbes Greifswald Germany

**Keywords:** spiders, overwintering, leaf litter, woody vegetation, noncrop habitat, landscape

## Abstract

**BACKGROUND:**

Successful overwintering habitats are critical for the long‐term survival of biocontrol agents in agricultural landscapes, promoting ecosystem services by preserving beneficial arthropods. Although it is known that predatory arthropods overwinter in leaf litter in fruit orchards, the potential of nonproduction woody vegetation in agricultural landscapes for supporting overwintering spiders is poorly understood.

**RESULTS:**

We compared spider assemblages overwintering in leaf litter of solitary trees, woody vegetation in line, and areal formations across three types of intensively used agricultural landscapes. We recorded 2502 overwintering spiders from 83 species and 20 families, with Linyphiidae being the most abundant. Assemblage composition and abundance were significantly influenced by landscape type, woody vegetation structure, adjacent land use, vegetation identity and litter weight. Linear and areal formations supported more spiders than solitary trees. Grass and leaf litter cover, as well as litter weight, affected both abundance and community composition. No significant differences were found in guild or ballooning traits across landscapes. Spider assemblages also differed between early and late winter, with higher abundance observed at the end of the season.

**CONCLUSION:**

Our study highlights that nonproduction elements of woody vegetation are important for the overwintering of spiders in agricultural landscapes. These findings are significant for landscape planning aimed at supporting ecosystem services and biodiversity conservation through the strategic integration of noncrop habitats. © 2025 The Author(s). *Pest Management Science* published by John Wiley & Sons Ltd on behalf of Society of Chemical Industry.

## Graphical abstract

Nonproduction woody vegetation provides crucial overwintering habitats for spiders, enhancing biocontrol services in agricultural landscapes. Incorporating these habitats into land management supports ecosystem services and biodiversity.

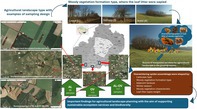

## INTRODUCTION

Agricultural intensification and habitat loss have caused a sharp decline in invertebrate populations globally.^1^ This biodiversity loss is largely a consequence of landscape homogenization driven by agrochemical use.^2^, ^3^, ^4^, ^5^ Historically, agricultural landscapes were diverse mosaics of croplands and semi‐natural habitats that supported high biodiversity.^6^, ^7^ However, this rich landscape has been simplified into larger, intensively managed agricultural areas,^8^ driven by demand for higher crop yields, mechanization, larger field sizes and reduced crop diversity.^9^ This landscape homogenization is evident at the field, habitat and landscape scales.^8^ The loss of biodiversity has led to a significant decline in ecosystem services, particularly biological pest control. As biodiversity decreases, predator populations also decline, disrupting natural pest control.^10^, ^11^

Noncrop habitats in agricultural landscapes play a vital role in providing arthropod predators with prey and refuge from field management activities.^12^ These habitats, including nonproductive woody vegetation, contribute to landscape heterogeneity and functional biodiversity.^13^ Despite past alterations, patches of noncrop habitats still exist in agricultural landscapes.^14^, ^15^, ^16^ These habitats are essential for overwintering invertebrates,^17^, ^18^ with successful hibernation being critical for arthropod survival.^19^ Providing suitable overwintering habitats promotes biodiversity and the survival of beneficial arthropods like pest predators.^20^, ^21^ Many natural enemies migrate to arable fields from noncrop habitats in spring.^22^ In particular, omnivorous invertebrate pest predators, such as spiders, are crucial for pest management.^23^ Spiders are dominant predators in agroecosystems^24^, ^25^ and are vital for biological control.^26^, ^27^, ^28^ Studies show that spider assemblages are shaped by landscape and local factors,^29^, ^30^, ^31^ but most research has focused on active‐season spider populations. Understanding the overwintering distribution of spiders is essential for their support as biocontrol agents. In Central Europe, spiders seek refuges to survive harsh winter conditions,^32^, ^33^ including tree bark, snail shells,^32^, ^34^ artificial shelters,^35^ and leaf litter.^36^, ^37^ Leaf litter in orchards is a key overwintering habitat for beneficial arthropods, with spiders being the most abundant group.^37^ Nonproductive elements of woody vegetation, such as solitary trees and tree groups, contribute to leaf litter production and provide suitable overwintering habitats for spiders in agricultural landscapes. This study is the first to explore the importance of these nonproductive elements and their environmental conditions in relation to overwintering spider communities in varying agricultural landscapes. Our findings can help inform landscape design to support spider populations and enhance ecosystem services in agricultural areas.

Therefore, the aim of our study was to determine and compare the importance of leaf litter of different nonproduction woody vegetation elements in three types of agricultural landscapes for the overwintering of spiders. Thus, we focused on addressing the following issues: (i) what spider assemblages overwinter in leaf litter of nonproduction elements of woody vegetation; (ii) how landscape factors form the overwintering spider assemblage; (iii) how local and microhabitat conditions form the overwintering spider assemblage; and (iv) are there differences in spider assemblages between the beginning and end of winter?

## MATERIALS AND METHODS

### Study area

**Figure 1:**
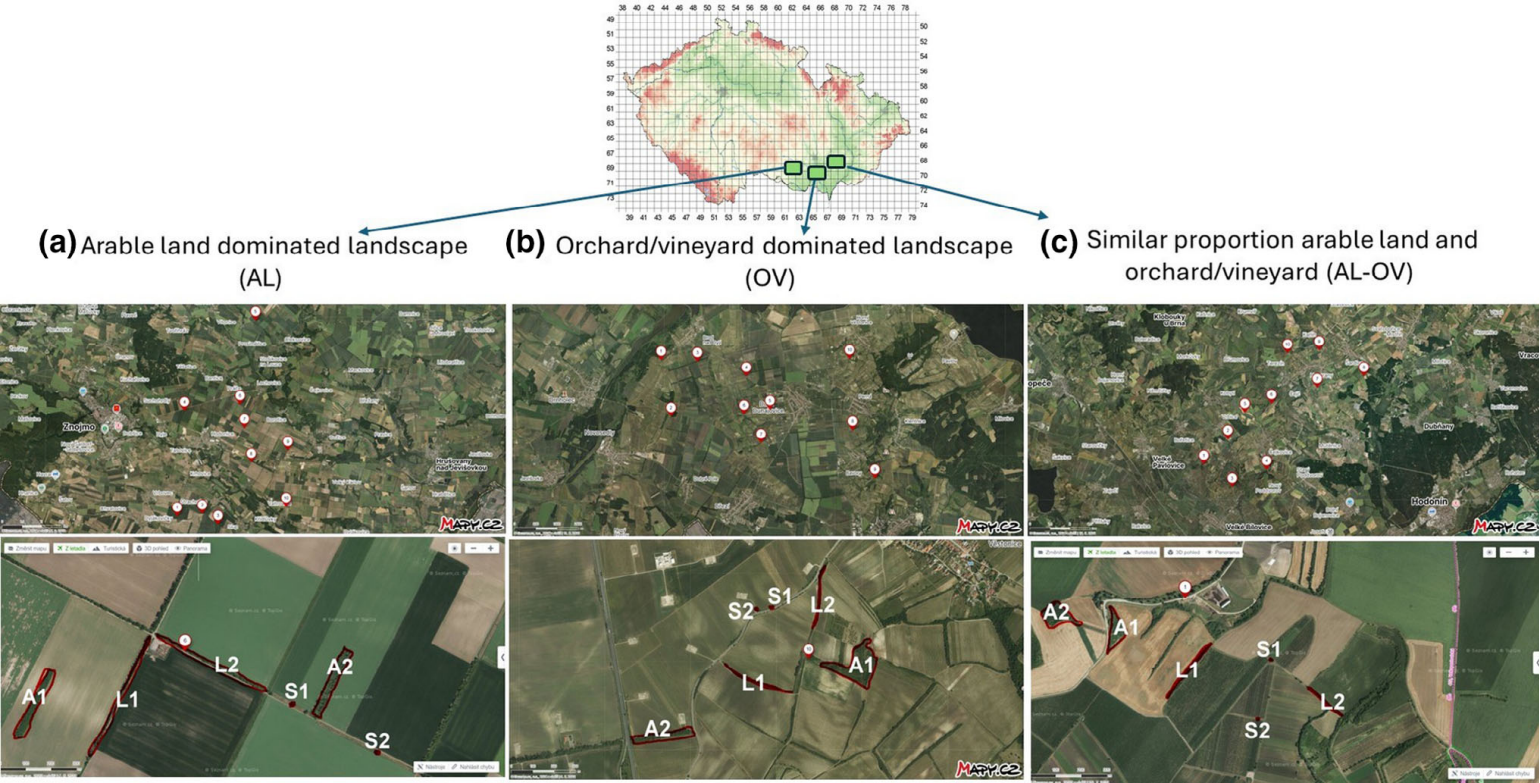
Sampling design of leaf litter in three types of intensively used agricultural landscapes of the Czech Republic. In each of the agricultural landscapes, 10 landscape windows were chosen. You can find the exact location of each point in (https://doi.org/10.6084/m9.figshare.28442840.v1.). The bottom three images are representative orthophotomaps of selected landscape windows. These images indicate typical landscape mosaics and land‐use in each investigated landscape, as well as leaf litter sampling in three types of woody vegetation formations as follows: A, aerial vegetation formation types; L, woody vegetation in line formations; S, solitary trees. The map of the Czech Republic was downloaded from BioLib.cz using a tool for drawing grid maps (https://www.biolib.cz/cz/tooltaxonmap/id1/). Orthophotomaps were downloaded from mapy.cz (https://www.mapy.cz). Maps were modified in faststone image viewer 7.7.

The investigation was carried out in three intensively used agricultural landscapes situated in South Moravia (Czech Republic, Central Europe; Fig. 1). The share of agricultural land reaches 60–70%.^38^ All three investigated regions are situated in a warm area of the Czech Republic which is characterized by a long, hot and dry summer, and a short spring and autumn. Winter is short, slightly warm, and dry, with a very short duration of snow. Investigated areas are located at an altitude of ≤300 m, an average annual temperature of 8–10 °C, a number of frost days of 90–130, a number of ice days of 0–40 and total precipitation in wintertime of 200–300 mm.^39^

The three investigated landscapes differ in land‐use, especially with the share of arable land and orchards/vineyards. The Znojmo region is characterized by arable land‐dominated landscape. In the investigated part of the Dolní Dunajovice region, we can find an orchard/vineyard‐dominated landscape. The Velkopavlovice region represents a landscape with a similar proportion of arable land and orchards/vineyards.^38^

### Sampling design and determination of spiders

In each of the three agricultural landscapes (each area reached 100 km^2^) 10 points were established, which were the centres of circular landscape windows. The maximum diameter of a landscape window was 1.0 km. To maintain as much independence in the observations as possible, the distance between points was kept at more than twice the diameter of the windows. There were established six nonproduction woody elements in each window: two solitary trees, two groups of trees in line formations and two groups of aerial woody vegetation. In total, it was proposed to obtain from each agricultural landscape 20 samples of leaf litter from solitary trees, 20 samples from line formations and 20 samples from nonlinear groups of trees at the beginning and end of winter 2022/2023 (Fig. 1). Leaf litters were sampled in dry weather when the air temperature did not exceed 10 °C.

Sampling was provided with a wooden square frame with a side length of 1 m. Each sampling locality, as well as the sample microhabitat inside the wooden square, was photographed to accurately determine the characteristics from which the sample was taken. Then leaf litter was placed in a plastic zip bag with a description of the sample. Each sample was weighted and sieved in the laboratory. Spiders were collected by hand and preserved in 70% ethanol.

The determination of spiders was performed according to the basic arachnological literature.^40^, ^41^, ^42^ Adult spiders were determined at the species level, whereas juveniles were determined at the higher taxonomic categories. The nomenclature follows the World Spider Catalogue.^43^

### Environmental variables and ecological characteristics of spiders

It can be assumed that several environmental variables will influence the choice of overwintering microhabitat. Therefore, we considered the type of agricultural landscape according to the land‐use: a landscape with a predominance of arable land (AL) (the Znojmo region), orchards/vineyards‐dominated landscape (OV) (the Dolní Dunajovice region), and a landscape with similar proportions of arable land and orchards/vineyards (AL‐OV) (the Velké Pavlovice region). As an environmental variable, the type of woody vegetation also was considered: solitary trees, trees in line formations and woody vegetation in areal formations. Detailed characteristics of each woody vegetation type also were taken into account as follows: for solitary trees–fruit tree, walnut tree, other tree; for trees in line formation–shrubs, mixed shrubs and trees; for areal woody vegetation–monoculture native trees, monoculture non‐native trees, polyculture. Adjacent land‐use also was recorded as follows: winter crop, ploughed land, vineyard/orchard and grassland. As for microhabitat characteristics, we calculated the percentage occurrence of leaf litter, bare ground, moss, grass and old grass in each orchard using the proportion that was recorded inside the sampling square. Using a Braun–Blanquet scale as inspiration,^44^ percentages were converted into categories 1–5 as follows: 5 = 75–100%, 4 = 50–75%, 3 = 25–50%, 2 = 5–25% and 1 = <5%. We also took into account the leaf litter weight of each sample.

Spiders are able to contribute to pest control in agricultural landscapes and are a significant part of food chains. It was found that some ecological characteristics of spiders, such as hunting strategy are closely connected with specific spider predatory behaviour (Michalko *et al*. 2018). The dispersal ability of spiders also is an important characteristic for their ability to reach and leave an overwintering habitat. According to diverse hunting strategies,^45^ spiders can be classified into seven categories called guilds: orb web weavers, sheet web weavers, space web weavers, ambush hunters, ground hunters, other hunters, and specialists. Dispersal ability was recorded as the ability to balloon^46^ in the following ways: very common (VC)—both juveniles and adults regularly balloon; less common (LC)—both juveniles and adults irregularly balloon; uncommon (U)—nonballooning adults with sometimes ballooning juveniles; data not available (NA). All the environmental variables and ecological characteristics are available at figshare repository at (https://doi.org/10.6084/m9.figshare.28442840.v1).

### Statistical analyses

In order to elucidate differences in spider assemblages between landscape types, woody vegetation types, and seasons, canonical correspondence analysis was used. Pairwise differences also were statistically tested using PERMANOVA implemented in the package vegan,^47^ with the Benjamini–Hochberg correction for multiple comparisons being applied.^48^ Each model included the tested variable and all other variables as covariates, with the tested variable always being the last term in the model to test the effect uniquely attributable to it. The overall effect of landscape type and woody vegetation formation type on abundance was tested using the Poisson regression. The absence of overdispersion was confirmed with the function dispersiontest() from the package AER^49^ and the subsequent likelihood‐ratio test. Follow‐up pairwise comparisons, where applicable, were performed using estimated marginal means implemented in the package emmeans ^50^ with the Tukey's HSD correction. The effect of detailed characteristics of woody vegetation was always analyzed separately for each woody vegetation formation type. The microhabitat texture variables were subjected to centered log‐ratio transformation before the analyses to account for their interdependence.

## RESULTS

### Which spider assemblage overwinter in leaf litter of nonproduction elements of woody vegetation

A total of 2502 spiders, belonging to 20 families, 82 genera and 83 distinct species were found overwintering in the leaf filler in the South Moravian agricultural landscape. Most individuals belonged to the families Linyphiidae (27.5%), followed by Dictynidae (16.5%), Thomisidae (10.7%), Clubionidae (8.9%), Philodromidae (8.3%), Gnaphosidae (6.2%) and Phrurolithidae (4%; see Research data material). Most spiders overwintered as juveniles (88%).

### How landscape factors form the overwintering spider assemblage

**Figure 2:**
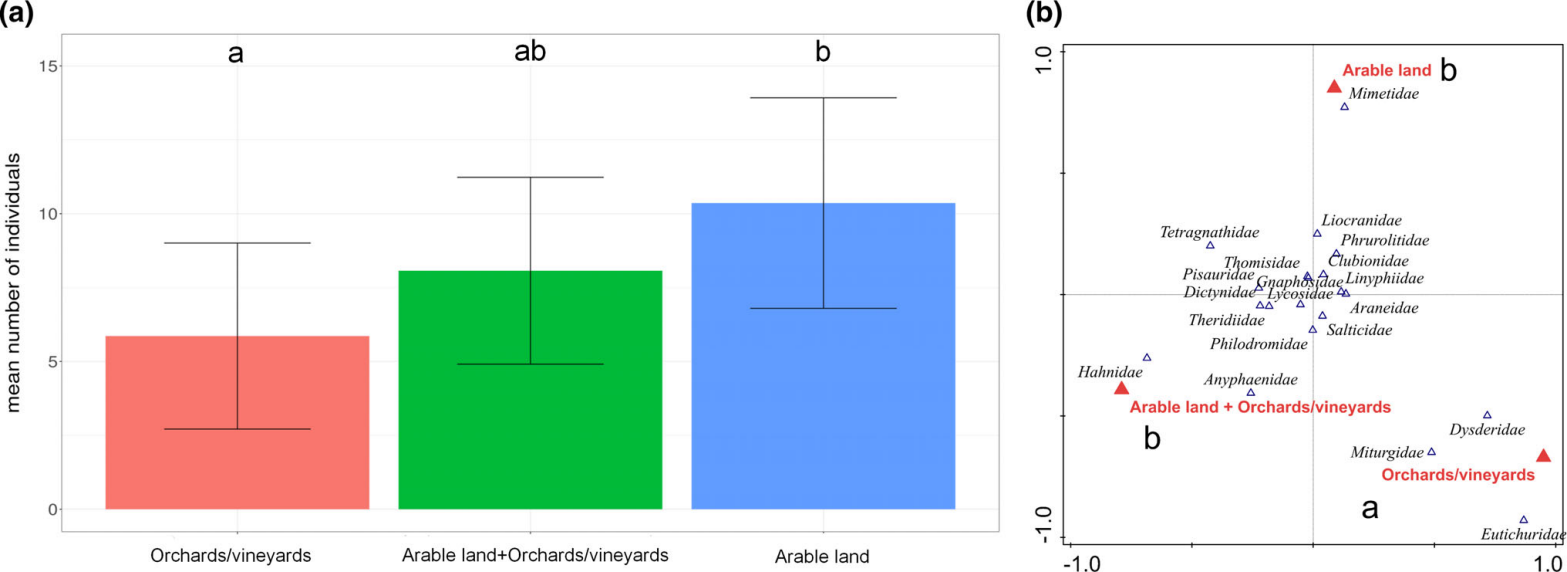
Comparison of mean number of individuals (a) and spider family assemblages (b) in the three types of agricultural landscapes according to land‐use. Agricultural landscapes sharing no letters significantly differ (*P* < 0.05). Significance of differences was tested based on estimated marginal means (not displayed). Bar heights indicate raw mean values.

A significant effect of landscape type on spider assemblages was observed. Specifically, significant differences in the mean number of individuals were found between OV and AL (*P* = 0.027) [Fig. 2(a)]. Spider morphospecies and family assemblages significantly differed between OV and AL‐OV (*P* = 0.009, *P* = 0.024, respectively) [Fig. 2(b)–only the effect on family assemblages is displayed]. Landscape type showed no significant effect on guild composition. Likewise, no effect was observed on the assemblage composition from the viewpoint of ballooning ability of individual species.

### How local and microhabitat conditions form the overwintering spider assemblage

**Figure 3:**
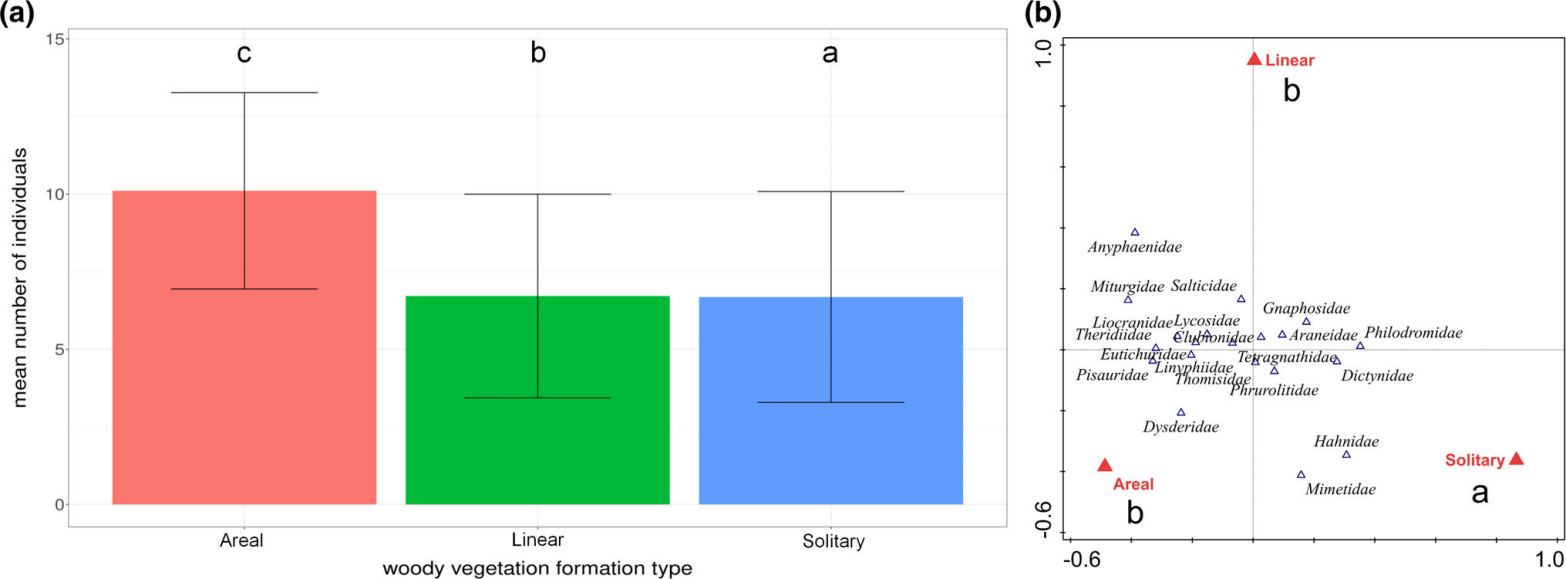
Comparison of mean number of individuals (a) and spider family assemblages (b) in the three types of woody vegetation formation types. Woody vegetation formation types sharing no letters significantly differ (*P* < 0.05). Significance of differences was tested based on estimated marginal means (not displayed). Bar heights indicate raw mean values.

Significant differences in the mean number of individuals were found between woody vegetation formation types, specifically between the solitary and linear (*P* < 0.0001), solitary and areal (*P* = 0.028), and linear and areal (*P* < 0.0001) types [Fig. 3(a)]. Significant differences in spider species and family assemblages were found between woody vegetation formation types, namely between the solitary and linear (morphospecies: *P* = 0.005, families: *P* = 0.003) and solitary and areal (species: *P* = 0.003, families: *P* = 0.003) types [Fig. 3(b)–only the effect on family assemblages is displayed].

**Figure 4:**
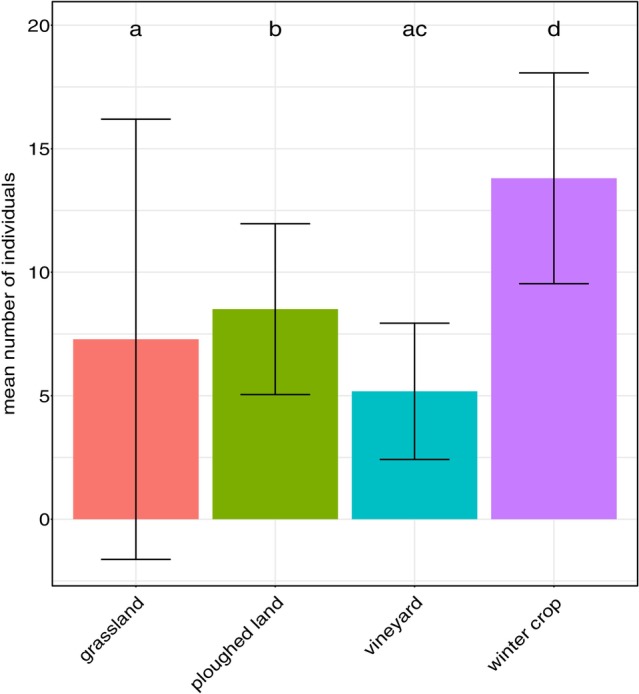
Comparison of mean number of individuals collected from leaf litter according to the adjacent land‐use. Adjacent land‐use categories sharing no letters significantly differ (*P* < 0.05). Significance of differences was tested based on estimated marginal means (not displayed). Bar heights indicate raw mean values.

A significant effect of adjacent land‐use on the mean number of individuals was observed, with all land‐use types differing from each other (*P* < 0.0001 in all cases), except for grassland‐vineyard (*P* = 0.98) (Fig. 4). The effect of adjacent land‐use on species and family assemblages as well as guild composition was nonsignificant (*P* = 0.088, *P* = 0.094, *P* = 0.351, for species assemblages, family assemblages and guild composition, respectively). The assemblage composition from the viewpoint of ballooning ability of species also did not differ significantly between the adjacent land‐use types (*P* = 0.804).

**Figure 5:**
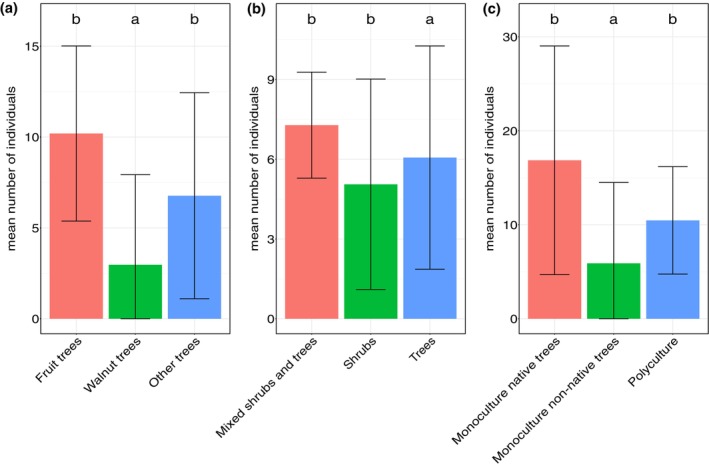
Comparison of mean number of individuals collected from leaf litter according to type of trees in solitary formation type (a), line formation type (b) and areal formation type (c). Significance of differences was tested based on estimated marginal means (not displayed). Bar heights indicate raw mean values.

Detailed characteristics of woody vegetation did not have a significant effect on any of the tested multivariate variables, but displayed a significant effect on the mean number of individuals. For the solitary formation type, a significant difference was observed between ‘walnut trees’ and ‘fruit trees’ (*P* = 0.009) and between ‘walnut trees’ and ‘other trees’ (*P* = 0.007) [Fig. 5(a)]. For the linear formation type, there was a significant difference between ‘mixed shrubs and trees’ and ‘trees’ (*P* < 0.0001) and ‘shrubs’ and ‘trees’ (*P* = 0.0003) [Fig. 5(b)]. In the case of the areal formation type, there was a significant difference between ‘monoculture native trees’ and ‘monoculture non‐native trees’ (*P* < 0.0001) and ‘monoculture non‐native trees’ and ‘polyculture’ (*P* < 0.0001) [Fig. 5(c)]. All other comparisons were nonsignificant.

Microhabitat texture showed a significant effect on the mean number of individuals, specifically the leaf litter and grass coverage (*P* < 0.0001), but the LR statistics were extremely small, at 4.55e‐13 and 2.27e‐13 for leaf litter and grass, respectively. There also was a significant effect of microhabitat texture on spider species assemblages and guild composition. In both cases, the effect was driven by the grass coverage (*P* = 0.018 for species, *P* = 0.025 for guilds).

**Figure 6:**
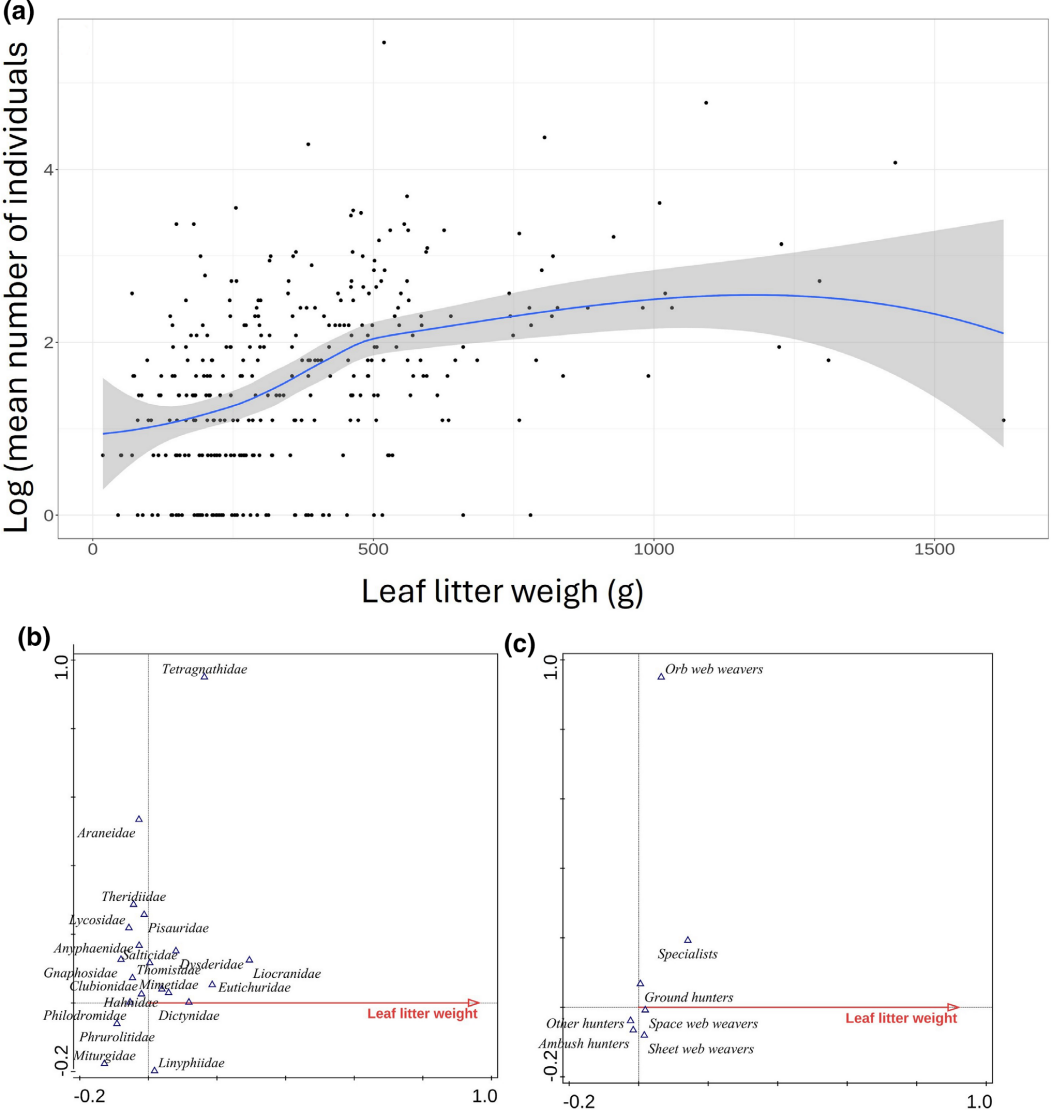
Effect of leaf litter weight (g) on mean number of collected individuals (a), families (b) and guilds (c).

The leaf litter weight had a significant effect on the mean number of individuals (*P* < 0.0001), species and family assemblages (*P* = 0.002, *P* = 0.001 for morphospecies and families, respectively), and guild composition [*P* = 0.001) (Fig. 6(a–c)].

### Are there differences in spider assemblages between the beginning and end of the winter?

**Figure 7:**
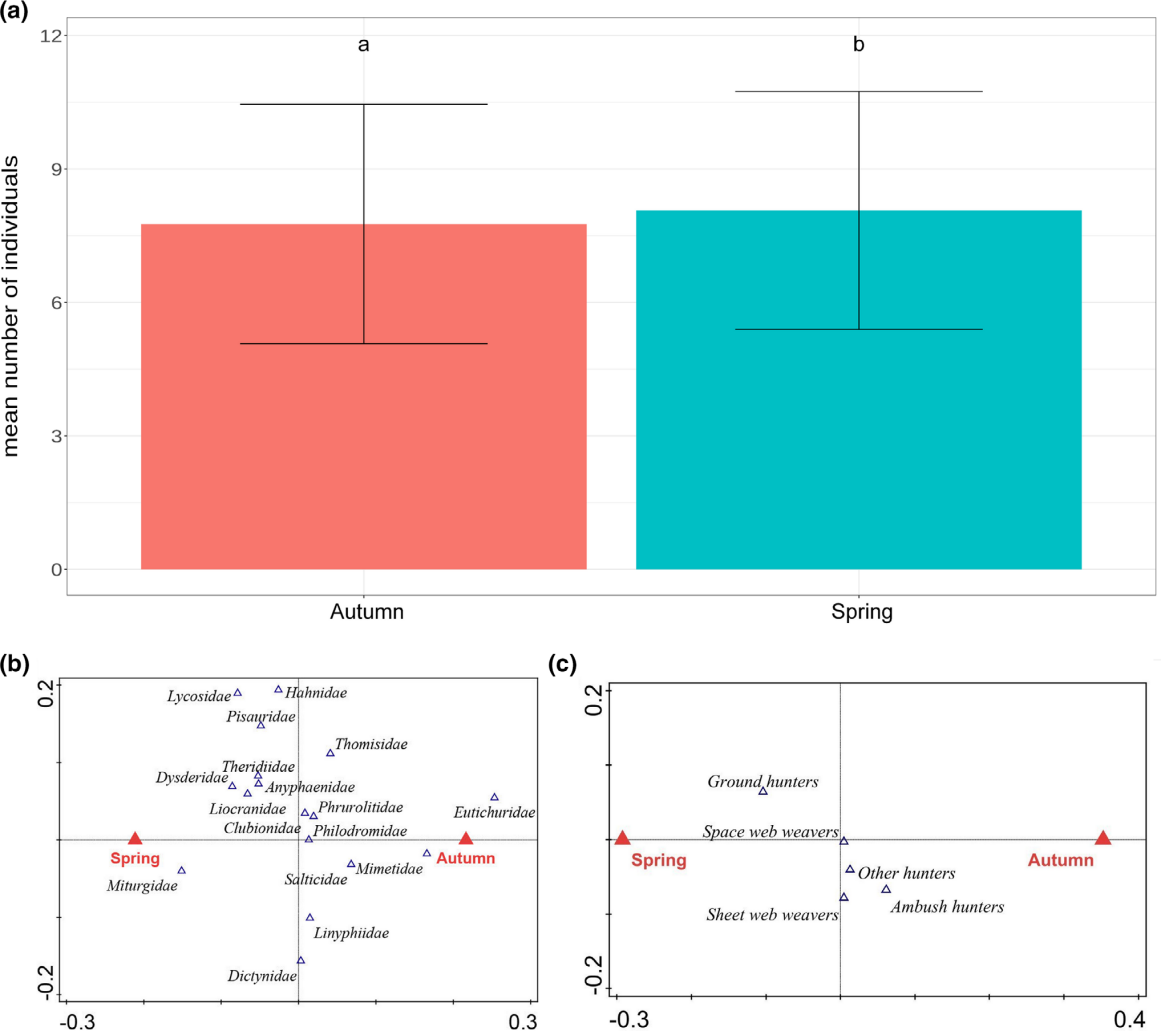
Comparison of mean number of individuals (a), families (b) and guilds (c) at the beginning and at the end of winter 2022/2023. Significance of differences was tested based on estimated marginal means (not displayed). Bar heights indicate raw mean values.

There was a significant effect of sampling season on spider assemblages. The mean number of individuals was significantly higher at the end of winter (*P* < 0.0001) [Fig. 7(a)]. The differences between seasons also were observed for morphospecies and family assemblages (*P* = 0.001 for morphospecies, *P* = 0.001 for families, respectively) [Fig. 7(b)], and guild composition (*P* = 0.001) [Fig. 7(c)].

## DISCUSSION

### Overview of overwintering assemblages in leaf litter of nonproduction elements of woody vegetation

Our study highlights the importance of leaf litter from nonproduction woody vegetation as an overwintering habitat for spiders in intensively used agricultural landscapes in South Moravia, Czech Republic. The most abundant families were Linyphiidae and Dictynidae, which together made up >40% of the total sample. Similar dominance of Linyphiidae has been reported in previous studies,^37^, ^51^ though the spider composition in orchards differed, likely as a consequence of intensive management practices and the thinner leaf litter layers. Our findings contrast somewhat with those of Mestre *et al*.,^51^ who identified Lycosidae, Linyphiidae and Gnaphosidae as the dominant families in semi‐natural habitats. Dictynidae, particularly the genera *Dictyna*, *Lathys* and *Nigma*, were commonly found in the leaf litter. These genera often coexist in the canopy.^52^ Thomisidae, which are typically less abundant in orchards and semi‐natural habitats,^37^, ^51^ also were present in leaf litter. Additionally, Clubionidae, a dominant group in leaf litter, were found in grassy field margins,^22^ although they were absent from orchard leaf litter.^37^

Most overwintering spiders were juveniles, similar to previous findings.^37^, ^53^ By contrast, Mestre *et al*.^51^ found that most overwintering spiders were adults. Differences in spider composition, age structure, and abundance may be a consequence of variations in sampling methods, timing, and landscape heterogeneity. Our study sampled overwintering spiders during October/December and February/March, whereas Mestre *et al*.^51^ sampled from February to June. Juveniles, which form a significant part of spider communities,^54^ are often excluded from ecological studies, but recent studies emphasize their importance.^55^ Excluding juveniles can distort ecological analysis, and our understanding of functional ecology and ecosystem services.

### Landscape effects on the overwintering spider assemblage

We found that spider abundance and assemblage composition varied among agricultural landscapes. Most spiders overwintered in leaf litter of AL‐dominated landscapes or AL‐OV. It is known that the distribution and abundance of arthropods depend on the properties of the wider landscape.^10^ Some studies report increased spider abundance in more diverse landscapes,^56^ yet these findings are likely to reflect patterns during the growing season, making them difficult to compare with our winter‐focused results.

We assume that overwintering spider communities respond differently to environmental conditions due to their specific composition. In the AL‐dominated Znojmo region, intensive soil disturbance in autumn is likely to force spiders to seek shelter elsewhere. Thanks to their excellent dispersal abilities,^57^ spiders may relocate into nonproductive woody vegetation. Although no significant difference was found between AL and AL‐OV landscapes, slightly fewer individuals were recorded in AL‐OV. The lowest numbers were found in OV‐dominated landscapes, which is likely to be because these offer more undisturbed overwintering sites directly within the agroecosystem.

We found an affinity of some spider families overwintering in leaf litter to landscape types. Dysderidae, Miturgidae and Cheiracanthidae were typically found in the OV landscape, whereas Anyphaenidae, Hahnidae and Philodromidae were found overwintering in leaf litter of woody vegetation in the AL‐OV landscape. It was previously found that landscape factors can have effects in shaping spider communities.^29^, ^30^, ^58^ Anyphaenidae and Philodromidae showed a previous affinity to OV landscapes.^54^, ^59^ Because juvenile spiders are highly mobile when searching for overwintering sites, and owing to the limited number of studies focused on overwintering spider assemblages, it is difficult to directly compare our findings with existing research. Most ecological studies examine spider communities during the growing season. However, we believe that overwintering communities are ecologically distinct, shaped by the specific requirements needed to survive the winter.

### Local effects on the overwintering spider assemblage

Our study found the highest mean number of individuals in areal woody vegetation. Several studies reported that local factors are very important for shaping spider assemblages.^30^, ^31^ Areal vegetation, being more robust, is easier for spiders to locate when seeking overwintering habitats. Its larger dimensions are likely support a diverse community of spiders–both those migrating from surrounding agricultural areas and those residing year‐round. Mestre *et al*.^51^ found that semi‐natural woody vegetation had a minor effect on spider community composition. We attribute the differences between our results and theirs to variations in timing, sampling methods and landscape matrices.

Our results show that overwintering spider assemblages are influenced by adjacent land use in terms of mean individual numbers. However, the absence of adjacent land‐use effects on overwintering spider families, morphospecies, guilds and dispersal traits may reflect reduced trophic activity, limited dispersal during winter and the overriding importance of local microhabitat conditions for winter survival.

Land use and management are key factors affecting spider abundance and species richness, especially in agroecosystems, although these effects were less noticeable in forests.^60^ Agricultural activities in adjacent crop fields are more disruptive than those in OV and grasslands, particularly at the end of the growing season when spiders seek overwintering habitats. We suggest that spiders escape disruptive environments by moving to nonproductive elements of woody vegetation to overwinter.

Detailed characteristics of woody vegetation influenced the mean number of sampled individuals. Leaf litter from solitary walnut trees hosted fewer individuals, which is likely to be a result of allelopathic substances in walnut leaves, which affect plant and insect interactions and alter microhabitat conditions.^61^ Additionally, we found differences in the abundance of spiders in linear formations, with variations between ‘mixed shrubs and trees,’ ‘trees,’ and ‘shrubs and trees.’ These differences may be linked to light conditions, as light intensities significantly impact species presence during the growing season.^62^ Large trees with tall trunks allow more light to reach the ground than shrubs, whose dense crowns block light.

### Microhabitat effects on the overwintering spider assemblage

Microhabitat characteristics, such as leaf litter and grass coverage, influenced the mean number of individuals, spider morphospecies assemblages and guild composition. Grass coverage was the main driver of changes in morphospecies and guilds, although the small effect sizes suggest the impacts may be biologically insignificant. The only study on overwintering spiders and microhabitat effects, found significant impacts on spider guilds but noted uncertainty about whether differences were caused by microhabitat or orchard position (edge *versus* inner areas), as these factors were correlated.^37^ Orchards, unlike the natural vegetation we studied, undergo management interventions, making comparisons difficult. Microhabitat characteristics are key to spider distribution and abundance in agricultural systems in the growing season.^63^ Further research is needed to understand the microhabitat conditions affecting overwintering invertebrates.

Leaf litter weight had a significant effect on the mean number of individuals, morphospecies, family, and guild assemblages, but did not significantly affect other analyses when included as a covariate. In forest ecosystems, arthropod abundance increases with deeper and more complex litter^64^, ^65^, ^66^ Leaf litter structure significantly affects spiders, particularly Linyphiidae in summer, but its impact reversed by autumn as spiders moved to overwintering habitats.^67^ In agricultural landscapes, leaf litter differs from that in forests, often being thin and making forest measurement methods unsuitable. Thus, leaf litter weight was used as a proxy for biomass, alongside cover characteristics, to better reflect conditions for spider presence.

### Are there differences in abundance, diversity and spider composition between the beginning and the end of winter?

Our study found that spider assemblages changed over the winter, with Linyphiidae, Dictynidae and Thomisidae more abundant at the beginning, and Gnaphosidae increased by winter's end. Spiders are highly adaptable, with varying winter strategies, from phenological phases to ballooning abilities.^57^ Some species, such as Philodromidae, Anyphaenidae and Clubionidae, remain active near 0 °C.^53^, ^68^ Environmental factors, such as the decomposition of plant litter, may further influence spider distribution through winter‐active arthropods.^33^ However, much is still unknown about the winter adaptations of other spider groups.

## CONCLUSIONS

Our study highlights the role of leaf litter of nonproduction elements of woody vegetation in intensively used agricultural landscapes for overwintering of spiders. We found that spider communities overwintering in the leaf litter of nonproductive woody vegetation elements are specifically shaped by landscape factors, formation and detailed characteristics of nonproductive woody vegetation and adjacent land‐use. We also found that spider assemblages changed during the wintertime. Preserving a variety of nonproduction elements of woody vegetation is crucial to providing suitable overwintering habitats to support spider communities, especially in agricultural landscapes with a large block of agronomically disturbed arable land, as the greatest number of overwintering individuals were discovered there. As biodiversity is an important factor contributing to ecosystem functioning, its loss can negatively impact ecosystem services across agricultural landscapes. Supporting overwintering polyphagous predators in landscapes can lead to earlier interventions against pests arriving to agroecosystems in early spring and overall support of biodiversity.

## CONFLICT OF INTEREST

The authors declare that they have no conflict of interest.

## Data Availability

The datasets generated during the current study are available in the https://figshare.com/s/175238e8e79a2fa74d3d?file=52468481, 10.6084/m9.figshare.28442840.
